# Mutational Signatures and Machine Learning for Risk Stratification of Acute Myeloid Leukaemia Based on Targeted Sequencing Data

**DOI:** 10.3390/cancers18121925

**Published:** 2026-06-12

**Authors:** Heba Elhaddad, Claudia Chiriches, Shuvro Prokash Nandi, Patrick van Eijk, Amanda Gilkes, Katie Watts, Amy Houseman, Charlotte S. Wilhelm-Benartzi, Oliver Gerhard Ottmann, Simon H. Reed, Martin Ruthardt

**Affiliations:** 1 Division of Cancer and Genetics, Section of Haematology, School of Medicine, Cardiff University, Cardiff CF14 4XN, UK; 2 Experimental Cancer Medical Centre (ECMC), School of Medicine, Cardiff University, Cardiff CF14 4XN, UK; 3 Clinical Pathology Department, Faculty of Medicine, Mansoura University, Mansoura 35516, Egypt; 4 Division of Cancer and Genetics, School of Medicine, Cardiff University, Cardiff CF14 4XN, UK; 5 Department of Cellular and Molecular Medicine, University of California San Diego, La Jolla, CA 92093, USA; 6 Centre for Trials Research, School of Medicine, Cardiff University, Cardiff CF14 4XN, UK

**Keywords:** acute myeloid leukaemia, induction chemotherapy, risk assessment, bioinformatics, machine learning, Random Forest, SMOTE balancing

## Abstract

**Background/Objectives:** To date, no validated scoring system can accurately predict the responses of acute myeloid leukaemia (AML) patients to induction chemotherapy (CTX). Current risk assessment relies on complex cytogenetic and molecular abnormalities and focuses on mutations in genes considered fundamental to leukaemogenesis. **Methods:** We performed bioinformatic analysis of targeted sequencing (TS) data from 111 genes in 1552 AML patients, focusing on mutational patterns derived from single-nucleotide variant (SNV) catalogues. The SNV catalogues were analysed using non-negative matrix factorisation (NNMF), a linear dimensionality-reduction approach, to extract risk-defining recursive signatures (RSs) and to distinguish responders from resistant patients following induction CTX. To enable patient-level prediction, we complemented NNMF with a Random Forest (RF) model. Given the class imbalance between responders and resistant cases, model performance was improved by applying the Synthetic Minority Over-sampling Technique (SMOTE) and by incorporating germline variants alongside somatic mutations. **Results:** NNMF-derived RSs captured clinically relevant structures in patients’ mutational profiles and clustered patients by treatment response, indicating that the diagnostic targeted sequencing data contain sufficient information for risk stratification and treatment response prediction. At the single-patient level, RF models incorporating balanced data and germline variation improved predictive performance compared with unbalanced somatic-only models. **Conclusions:** These findings demonstrate that machine learning applied to targeted sequencing data can extract clinically informative mutational structures and improve risk stratification in AML, supporting its potential integration into precision treatment decision-making.

## Simple Summary

To date, it has been impossible to accurately predict the responses of acute myeloid leukaemia (AML) patients to induction chemotherapy (CTX). Currently, risk assessment is complicated and not precise. We tested different bioinformatics tools and datasets to assess whether it is possible to predict the risk and response to induction CTX exclusively from patients’ genomic sequencing data at diagnosis. To confirm our results, we compared our findings with current risk assessments. We found that our bioinformatics tools could improve risk assessment for AML patients by better predicting their response to induction CTX. Better risk stratification of AML patients could spare patients deemed to be resistant to therapy the toxic effects of drugs they would not respond to.

## 1. Introduction

Acute myeloid leukaemia (AML) is characterised by impaired terminal differentiation and aberrant self-renewal of hematopoietic progenitor cells in the bone marrow (BM) [1,2]. AML is a malignant disease mainly of the elderly. Its standard intensive induction chemotherapy (CTX) aims to induce complete remission (CR). It is mainly based on the use of anthracyclines and cytarabine. Induction CTX exposes the patient to life-threatening toxicity, as the high early death rate and treatment-related deaths show [3,4,5].

Although the response rate is high (~80%), there are still patients who are resistant to induction CTX. Therefore, knowing whether the induction CTX is effective would be fundamental. Given that the failure of induction CTX is associated with adverse clinical outcomes to subsequent therapies and poor prognosis, such knowledge could protect patients from avoidable toxicity and potentially improve overall outcomes [3].

To date, no validated scoring system can accurately predict patients’ responses to induction CTX [6]. Most prediction approaches are complicated, time-consuming, expensive, and utilise various cytogenetic and molecular abnormalities. Walter et al. analysed 4601 AML patients using multivariable clinical, cytogenetic, and molecular predictors to estimate resistance to induction CTX, achieving an area under the curve (AUC) of 0.64–0.69 [7]. Ng et al. developed a 17-gene leukaemia stem cell (LSC17) gene-expression signature using stemness-associated transcriptional profiles and reported concordance statistics (C-statistics) ranging from 0.65 to 0.80 for risk and outcome prediction [8]. However, these approaches rely on integrating multiple clinical, molecular, or gene-expression parameters and require complex predictive models. In addition, outcome measures based on C-statistics may be influenced by events such as death and relapse, potentially introducing bias into model performance assessment [8,9].

Therefore, we sought to determine whether genomic mutational patterns alone contain sufficient information to predict treatment response in AML, independent of conventional clinical parameters. Addressing this question requires analysing large-scale genomic datasets and employing computational approaches capable of extracting biologically meaningful information from high-dimensional mutational data. The increasing number of patients from whom sequencing data are available continuously augments the system’s dimensions. In order to obtain information from these mutational data, a reduction in complexity through Linear Dimension Reduction (LDR) algorithms, such as non-negative matrix factorisation (NNMF), is performed [10,11,12]. NNMF enables the extraction of mutational signatures (MS) from somatic mutations in patients’ genomic data, reflecting the tumour’s mutational history and the potentially underlying mechanisms of tumour development [10].

We hypothesised that if NNMF can extract the biological history of a cancer from its somatic mutational profile, then the patient’s genome may also contain information regarding future disease behaviour. In AML, the primary therapeutic goal following diagnosis is the achievement of CR through induction CTX. Therefore, to improve AML risk stratification, we investigated whether genomic features could predict response to induction CTX and provide prognostic information independent of conventional clinical parameters. While clinical variables such as white blood cell counts and organ function markers are routinely used for patient assessment, they primarily reflect the patient’s current clinical condition. In contrast, genomic features directly represent the underlying biology of the disease and may therefore provide a more objective basis for predicting treatment response and patient outcome.

Genomic investigations for clinical purposes are often limited to the targeted sequencing (TS) of driver mutations in genes deemed relevant to disease pathogenesis. TS is faster and more cost-effective than whole-genome or whole-exome sequencing (WGS/WES). For our study, we had access to TS data for 111 genes from 1552 AML patients in the AML-NCRI cohort [13], which served as our primary dataset for bioinformatic analyses. We also had access to the TS genomic data of the AML-SG cohort [14,15], as well as WGS/WES data from The Cancer Genome Atlas (TCGA) [16]. We wanted to determine whether it is possible to extract sufficient information from TS to answer the questions using bioinformatics, without the need for clinical parameters. First, we used NNMF to create a matrix that combined the dataset with biological features, e.g., the response to induction CTX. NNMF extracted patterns of information in the form of recursive signatures (RSs), which represent the encoded information about biological behaviour within a given dataset. In addition, we applied Random Forest (RF) analysis to our TS dataset. RF is a widely used machine learning (ML) algorithm for predicting cancer-related outcomes, including disease progression and overall survival (OS) [17,18]. Unlike NNMF, RF is not limited to somatic mutational data and can incorporate a broader range of genomic data.

## 2. Materials and Methods

### Molecular Annotation

**Datasets:** The sequencing data of 111 genes of a cohort of 1552 AML patients from the AML-NCRI study group (including patients from AML14, AML15, AML16, and AML17) were retrospectively analysed. All patients in the AML-NCRI dataset underwent intensive induction CTX, and the CR rate was approximately 80%, matching rates reported in the literature [19]. Of the patients in the CR group, 47% relapsed during follow-up. The TS panel comprised the same 111 myeloid cancer-associated genes as previously described [14,15]. All samples from the NCRI trials were processed, sequenced, and annotated using a standardised pipeline, and raw data were deposited in the European Genome-Phenome Archive (EGA; accession EGAS00001000570) [13].

We derived the AML-SG dataset from the data repository [15] and the European Genome-Phenome Archive (EGA; accession EGAS00001000275). The AML-SG cohort included patients from HD98A, HD98B, and 07-04 studies [15]. We obtained the data of The Cancer Genome Atlas (TCGA) AML patients from the TCGA website [16].

**Preparation of custom capture libraries for sequencing:** Peripheral blood (PB) or BM mononuclear cells (MNCs) were collected from all patients at the time of diagnosis. Genomic DNA was extracted. Custom RNA baits were designed (SureSelect, Agilent, UK) to complement all coding exons of the 111 genes and genome-wide SNP probes. DNA was then fragmented to an average insert size of 145 bp and subjected to Illumina DNA sequencing library preparation using the bravo automated liquid-handling platform. Six PCR cycles were used for individual sample indexing. The hybridisation of equimolar pools of 16 libraries with the custom RNA baits was done. The sequencing of enriched pools from 96 cases was performed on the Illumina HiSeq Genome Analyser using a 600× median-coverage protocol [13].

**Sequencing data alignment:** Raw sequence data (BAM files) were aligned to the human genome (NCBI build 37) using BWA [20]. Unmapped reads, PCR duplicates, and reads mapping to regions outside of the target region were excluded from the analysis. Bedtools^®^coverage v2.15.05 was used to check the coverage depth at each base, followed by the exclusion of genes with median target coverage <20× and samples with median overall coverage <50× [13].

**Variant Calling on AML-NCRI TS for somatic mutations:** For each sample, single-base somatic substitutions (SBSs) were called using the algorithm CaVEMan (Cancer Variants through Expectation Maximisation) [21]. CaVEMan compares the sequencing data of each sample to an unrelated normal sample and calculates the probability of mutation at each base-pair position locus.

**Variant Calling for predisposition data (Germline + somatic mutations):** After decryption and decompression, the following steps were applied independently to each BAM file/patient sample: (I) Variant calling using Deep Variant to produce a VCF file for each patient; (II) Variant annotation using VEP to generate the final annotated VCF files for patients.

After generating Excel files for somatic mutations and VCF files for predisposition data, data tidying and preparation, as well as clinical data joining, were performed in R 4.3.1 and R Studio using several packages, including dplyr, tidyverse, janitor, and ggplot. It was done independently for each data type. All variants, even those of unknown significance, meaning those outside the range of frequent driver variants in genes with known oncogenic variants, variants in genes whose role in myeloid disease is not yet established, or variants presenting uniquely in the dataset, were included in the study as part of the mutational data (for a workflow, see Supplementary Figure S1).

**Generation of the mutational matrix:** Patients’ SBS mutational data were used to generate the mutational matrix after excluding insertions and deletions (indels). The SBSs were processed in the form of trinucleotides that included the 5′ and 3′ nucleotides adjacent to each identified SBS. This trinucleotide format was called the inclusion of SBSs within their trinucleotide context. The collection of the trinucleotide context of the six main identified SBSs resulted in 96 different trinucleotide SBSs. The presence and the quantity of each of the 96 trinucleotide SBSs in each patient’s genomic data were assessed, and the relative contributions of the different 96 trinucleotide SBSs to the constitution of the mutational profile (catalogue) of a patient were estimated and collected to constitute the 96 SBS mutational catalogue (96 trinucleotide SNV catalogue) of the patient. After that, the mutational catalogues of all patients or a group of patients sharing common clinical features were compiled to generate the 96 trinucleotide SNV mutational catalogue for the dataset or the patient group, respectively. These catalogues formed the basis for the mutational matrix that NNMF utilised to extract the RSs.

**NNMF for the de novo extraction of RSs:** NNMF extracted de novo RSs from mutational matrices. The R package ‘MutationalPatterns’ R/Bioconductor package was used for the extraction of MS/RS by NNMF [10]. The factorisation rank survey based on the cophenetic coefficient and rss (Residual Sum of Squares) was assessed, and consensus heatmap clustering was generated. The cophenetic coefficient and rss were used to determine the optimal number of signatures that could be extracted from each cohort. The optimal number of signatures was defined as the rank with the highest cophenetic coefficient that, when increased by one, did not result in a substantial reduction in rss (Supplementary Figure S2).

**Cosine Similarity and the Reconstruction of Patient Group Mutational Profile:** The similarity of the patient groups’ mutational profiles to known Cosmic-SBS MSs (COSMIC SBS signatures) was assessed by the Cosine Similarity analysis [11]. The similarity between the patient groups’ mutational profiles and the 95 known Cosmic-SBS MSs indicated the biological processes that may contribute to the mutational patterns observed in these groups [11]. The contribution of the Cosmic-SBS MSs to the reconstruction of the patient groups’ mutational profiles was assessed to confirm the results of the cosine similarity analysis. This approach was useful to link defective DNA Damage and Repair (DDR) mechanisms or other mutational processes to the biological behaviour of patient groups. This Euclidean-norm residual minimisation approach is used to reconstruct the mutational profile of a sample using either known or de novo-extracted mutational signatures. The ‘MutationalPatterns’ R/Bioconductor package integrated this algorithm from another R package called ‘pracma.’ Similarly, the de novo-extracted RSs were analysed and compared with the mutational profiles of the patient groups and with Cosmic-SBS MSs to stratify patients according to their response to induction CTX and to identify potential mutational processes contributing to the outcomes observed in each group. As the cosine of two non-zero vectors, such as A and B, can be measured, and the cosine similarity cos(θ), for these A and B vectors, will be the measure that calculates the cosine distances of the angle between them. The elements A and B are non-negative, and the cosine similarity has a range between 0 (independent matrices) and 1 (same matrices).

**Random Forest (RF):** The supervised ML algorithm RF is a bagging-type ensemble of decision trees that trains several trees in parallel and uses the majority vote of the trees as the final decision of the RF model. The combination of hundreds of decision tree models reduces variance and bias, enabling RF to produce more robust classification.

RF analysis was performed in R 4.3.1 and R Studio using the “caret,” “rpart,” “rpart.plot,” and “randomForest” packages. The previously generated individual mutational catalogues for the patients were joined to form a single (non-pooled) matrix containing the frequency of each of the 96 trinucleotide SNVs for each patient. Clinical data on patients’ responses to induction CTX were added to the matrix. RF then utilised this matrix to predict patient outcomes based on combinations of specific SNVs that were characteristic of each response group. To perform RF analysis, the matrix was randomly split into a 70% training set and a 30% test set. The RF model fit was then generated by training 1000 trees using the combined clinical/SNV matrix of the training set. The fit is supposed to be trained to identify the SNV patterns unique to each response group. Then it should repeatedly train itself to extract the most important variables for predicting the response group in new patient sets. The RF model fit was then used to predict the test set outcomes (a set of patients not previously encountered by the algorithm). A confusion matrix was constructed to compare the RF-predicted responses of the test set with the actual patient responses in order to assess model performance in terms of sensitivity, specificity, positive predictive value, negative predictive value, accuracy, and balanced accuracy.

RF analysis performed on data elaborated from BAM files (somatic and germline mutations) was done the same way as the previous analysis, except for the product matrix preparation, which included different steps starting from the VCFs elaboration to create new SNV mutational catalogues for each patient, including all the patients’ somatic and germline mutations, and then finally joining the patients’ catalogues to form the single non-pooled matrix that RF can use.

**Synthetic Minority Over-sampling Technique (SMOTE):** To address class imbalance, we applied SMOTE, a widely used data-balancing technique with RF. SMOTE introduces synthetic examples by interpolating between several minority-class instances within a defined neighbourhood [22]. This process utilises the nearest neighbours and considers each minority sample. After that, synthetic examples are produced along the line segments connecting to all k nearest neighbours. In addition, the majority-class instances are downsampled to achieve a more balanced dataset. Compared with simple oversampling with replacement, SMOTE has been shown to achieve improved performance under the Receiver Operating Characteristic (ROC) curve [23]. In our study, SMOTE was applied using the DMwR package to balance the training set after data splitting [24], as RF models trained on an unbalanced dataset were biassed toward the majority class. Data balancing using SMOTE was therefore essential to enhance the model’s predictive performance.

## 3. Results

### 3.1. TS of the AML-NCRI Patients Overlapped with the TS of the AML-SG and the WGS/WES of TCGA

As an initial step, we aimed to confirm that our TS data are comparable to WGS or WES data. We therefore evaluated whether mutational data derived from the TS of 111 genes in 1552 AML-NCRI patients or in 1540 AML-SG patients contained a comparable amount of genomic information relative to the WGS/WES data from 200 TCGA AML patients. Specifically, we analysed the mutational burden in: (I) the TS of AML-NCRI patients; (II) the WGS (*n* = 50) and the WES (*n* = 150) of the 200 TCGA AML patients [16]; and (III) the TS of AML-SG patients. Regarding AML-SG data, analyses were restricted to patients with available clinical data, reducing the AML-SG dataset from 1540 to 1077 patients [14,15].

**Figure 1:**
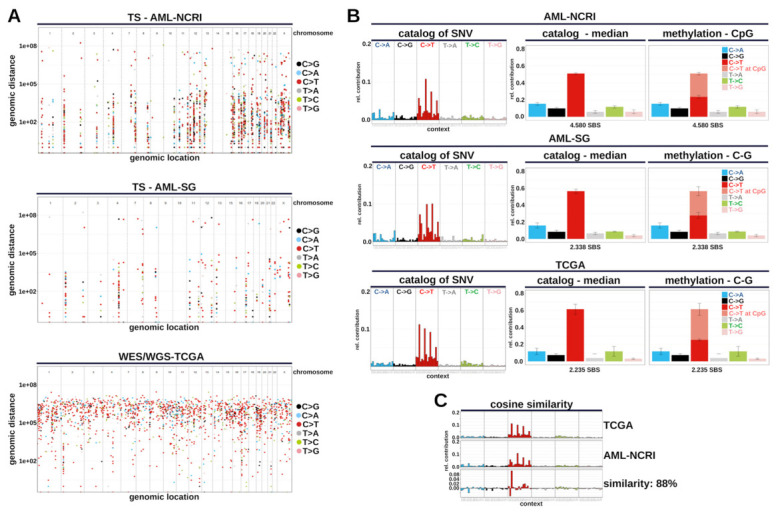
Comparison of single-nucleotide variant (SNV) mutational data derived from targeted sequencing (TS) and whole-genome sequencing/whole-exome sequencing (WGS/WES) data. (**A**) Rainfall plots of SNV catalogues derived from TS data of AML-NCRI and AML-SG cohorts, and from WGS/WES data of TCGA AML cohort, highlighting differences in mutation density and spatial clustering patterns. (**B**) SNV catalogues and median mutation distributions stratified by ± CpG island methylation status. (**C**) Cosine similarity between SNV catalogues of TCGA and AML-NCRI cohorts demonstrates the degree of concordance between mutational patterns derived from WGS/WES and TS platforms.

First, rainfall plots were generated, revealing a higher number of mutations in the AML-NCRI dataset compared with the AML-SG dataset, likely reflecting the larger patient cohort. The distribution of SNV mutations in the TS datasets (AML-NCRI and AML-SG) was comparable across both rainfall plots. In contrast, SNV mutations in the TCGA, WGS, and WES data were distributed across all chromosomes (Figure 1A).

Next, we investigated the mutational patterns of the SNV catalogues of the WGS/WES TCGA dataset [16] and the two TS datasets, AML-NCRI and AML-SG [14,15]. Thus, we created pooled mutational catalogues for each dataset by combining all patients’ SBSs in their trinucleotide context, generating the 96 trinucleotide SNV catalogues. The two TS datasets, although differing in the number of SNVs analysed (AML-NCRI-4580; AML-SG-2338), did not differ in SNV patterns within the catalogues, confirming their similarity (Figure 1B). In the TCGA dataset, the SDs of the T > A and T > C columns indicated a wide range of SNV frequency variability (Figure 1B). In contrast, the small SD for the other columns indicated only minimal SNV variation among the 200 patients. Nevertheless, the TCGA catalogues reproduced the pattern of somatic mutations observed in the TS datasets, with an 88% cosine similarity between the SNV catalogues of the TCGA and the AML-NCRI datasets (Figure 1C).

These findings indicated that the TS of 111 genes in the AML-NCRI (1552 patients) and AML-SG sets (1077 patients) provided a similar pattern of genomic data as that in the WGS/WES of the 200 TCGA AML patients, supporting its suitability for in-depth bioinformatic analyses.

### 3.2. Risk Stratification of AML Patients Using MS Analysis of WGS/WES and TS Data

We were looking for a bioinformatic tool that would allow us to make a risk assessment and predict therapy response in AML patients based exclusively on genomic data. We wanted to understand whether different datasets contain the information needed for such analyses. Therefore, we applied the LDR algorithm, NNMF, to extract signatures from the genomes of patients with a single cancer subtype, AML.

**Figure 2:**
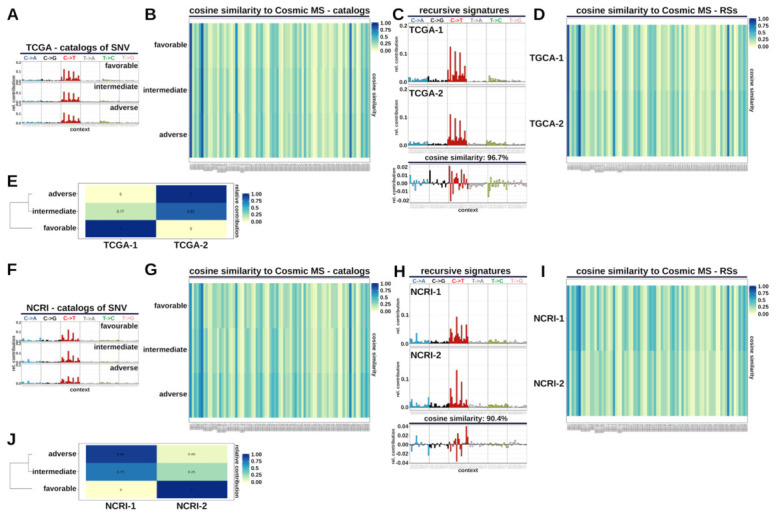
Risk stratification by NNMF MS analysis of TCGA WGS/WES dataset and AML-NCRI TS dataset. (**A**) SNV catalogues of TCGA patients stratified according to risk classification, illustrating differences in mutational patterns across risk groups. (**B**) Cosine similarity of SNV catalogues to Cosmic-SBS MSs, showing possible related mutational processes. (**C**) RSs (TCGA-1 and TCGA-2) extracted by NNMF and their mutual cosine similarity. (**D**) Cosine similarity of TCGA-1 and TCGA-2 to Cosmic-SBS MSs, highlighting correspondence with known mutational processes. (**E**) Contribution of TCGA-1 and TCGA-2 to the reconstruction of the mutational catalogues of TCGA risk groups, demonstrating their explanatory capacity. (**F**) SNV catalogues of AML-NCRI patients stratified according to risk classification, showing variability in mutational patterns. (**G**) Cosine similarity of SNV catalogues to Cosmic-SBS MSs in AML-NCRI cohort. (**H**) Recursive signatures (NCRI-1 and NCRI-2) extracted by NNMF and their mutual cosine similarity. (**I**) Cosine similarity of NCRI-1 and NCRI-2 to Cosmic-SBS MSs. (**J**) Contribution of NCRI-1 and NCRI-2 to the reconstruction of the mutational catalogues of AML-NCRI risk groups, showing comparable risk-group clustering to that observed in the independent TCGA cohort.

NNMF analysed the WGS/WES data from 200 TCGA AML patients. As a first step, we grouped these patients according to their risk classification into favourable, intermediate, and adverse. Next, we created the SNV catalogues by including the 5′ and 3′ nucleotides flanking each SBS to form the 96 trinucleotide SNV pooled catalogue for each risk group (Figure 2A). When compared to the Cosmic-SBS MSs, all SNV catalogues showed a high cosine similarity to the Cosmic-SBS MSs 1, 6, 15, and 87. In contrast, there were differences between the three risk groups regarding Cosmic-SBS MSs 12, 16, 26, and 37 (Figure 2B). These catalogues were used to construct the matrix from which NNMF extracted two novel signatures, the recursive signatures (RS) TGCA-1 and TGCA-2, which most effectively distinguished the three patient risk groups. (Figure 2C, Supplementary Figure S2A). Although the two RSs exhibited more than 96% similarity (Figure 2C), they showed differences in their cosine similarity to Cosmic-SBS MSs, particularly with respect to Cosmic SBS 11, 25, 32, 42, and 84. These differences highlight the uniqueness of the RSs and underscore the significance of the subtle variations between them (Figure 2D).

To determine the clinical significance of TCGA-1 and TCGA-2, we investigated their contribution in reconstructing the mutational SNV catalogues of the three clinically defined risk groups of AML. We found that patients in the adverse- and intermediate-risk groups clustered together and were characterised by a high contribution from TCGA-2. In contrast, patients in the favourable-risk group formed a separate cluster, with TCGA-1 as the sole contributor to their mutational catalogue (Figure 2E).

To evaluate whether NNMF could cluster AML patients into their respective risk groups using TS data, as observed with WGS/WES data, we performed the same analysis on the AML-NCRI TS dataset. Patients (*n* = 1552) were stratified into favourable-, intermediate-, and adverse-risk groups, and pooled SNV catalogues were generated accordingly (Figure 2F). When SNV catalogues were compared to the Cosmic-SBS MSs, all groups’ catalogues showed a high cosine similarity to the Cosmic-SBS MSs 1, 6, 15, and 87. In contrast, there were differences between the three groups regarding Cosmic-SBS MSs 19, 26, and 56 (Figure 2G). NNMF analysis (Supplementary Figure S2B) identified two novel RSs, NCRI-1 and NCRI-2, that distinguished the three risk groups in a pattern comparable to that observed in the TCGA analysis (Figure 2H, I). Although NCRI-1 and NCRI-2 showed 90.4% similarity, their relative contributions to the mutational burden of the risk groups led to the clustering of the adverse and intermediate groups together, with NCRI-1 making a substantial contribution to the mutational catalogues of both groups. In contrast, patients in the favourable risk group formed a distinct cluster with an exclusive contribution of NCRI-2 to their catalogue (Figure 2J).

AML patients within the same risk group, despite sharing a clinical classification, display substantial molecular and cytogenetic heterogeneity. The fact that the RSs were consistently able to distinguish the favourable-risk patients from those with intermediate- and adverse-risk strongly indicates that patients’ genomes at the time of diagnosis contain hidden information about the disease progression, which underscores the considerable potential of hidden genomic data to uncover clinically relevant insights beyond conventional risk assessment.

### 3.3. The Changes in the Risk Assessment from ELN2017 to ELN 2022 of AML-NCRI Were Recapitulated by the NNMF Analysis

We based our initial investigations on the 2017 risk assessment of the European Leukaemia Net (ELN) [25]. In 2022, ELN updated the risk classification of AML [26]. To evaluate the impact of the updated classification on our bioinformatic analysis, patients were reclassified according to the ELN 2022 risk stratification criteria [27], and NNMF analysis was repeated using the reclassified cohort. The resulting changes in risk assignment between ELN 2017 and ELN 2022 were then compared with the corresponding changes in the NNMF results.

For reclassification, we first assessed risk-defining cytogenetic abnormalities. Favourable-risk abnormalities included inv(16), t(8;21), and t(15;17); adverse-risk abnormalities comprised t(6;9), 11q23 rearrangements, t(9;22), inv(3), t(3;3), t(3q26), del(5q), del(7q), 17p abnormalities, complex karyotype, and monosomy, while t(9;11) was classified as intermediate-risk. Patients without these abnormalities were evaluated for TP53 mutations, with TP53-mutated cases assigned to the adverse-risk group. TP53-wild-type patients were then stratified by FLT3-ITD status. FLT3-ITD-positive patients were assessed for myelodysplasia-related (MR) gene mutations (SRSF2, SF3B1, U2AF1, ZRSR2, BCOR, EZH2, STAG2, ASXL1, RUNX1), with mutated cases classified as adverse-risk and non-mutated cases as intermediate-risk. Among FLT3-ITD-negative patients, NPM1 mutations were defined as a favourable-risk. NPM1-wild-type patients harbouring in-frame bZIP domain mutations in CEBPA were also classified as favourable. In contrast, CEBPA-negative patients with MR gene mutations were classified as adverse, and those without MR mutations as intermediate-risk [27] (for an in-depth comparison of the effect of the ELN 2022 criteria on the 1552 AML-NCRI patients’ classification, see Supplementary Figure S3A–C).

**Figure 3:**
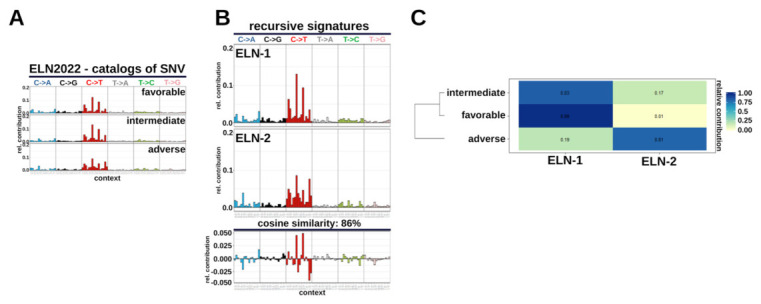
The influence of ELN 2022 classification on risk assessment by NNMF. (**A**) SNV catalogues of AML-NCRI patients grouped according to ELN 2022 risk categories, illustrating differences in mutational pattern distribution across risk groups. (**B**) RSs ELN-1 and ELN-2 extracted by NNMF and their cosine similarity. (**C**) Contribution of ELN-1 and ELN-2 to reconstruction of mutational catalogues of AML-NCRI patient risk groups, demonstrating clustering of intermediate-risk group with favourable-risk group rather than with adverse-risk group under ELN 2017 classification.

Next, we constructed SNV catalogues for the AML-NCRI patient risk groups, classified according to the ELN 2022 criteria, to create the NNMF matrix (Figure 3A).

NNMF extracted two novel RSs, ELN-1 and ELN-2, which showed 86% similarity (Figure 3B). Notably, these signatures altered the clustering pattern of the patients: the intermediate- and favourable-risk groups clustered together, whereas the adverse-risk group formed a distinct cluster (Figure 3C). This change in risk-group clustering aligns with the recently reported impact of ELN 2022 risk-group reclassification on AML patient outcomes. [26].

These findings show that NNMF can accurately capture changes in mutational data across patient groups and cluster them accordingly, supporting its use in predicting patient outcomes and identifying potentially associated mutational processes.

### 3.4. RSs Can Differentiate AML Patients According to Their Response to Induction CTX

The ability of NNMF to extract information relevant to AML risk classification prompted us to investigate whether NNMF could predict responses to induction CTX from AML-NCRI TS data, independent of the patients’ assigned risk groups.

First, we excluded 107 patients who died before day 28 after the beginning of therapy (the time point where the CR was assessed) because these early deaths (ED) could not be reliably classified.

**Figure 4:**
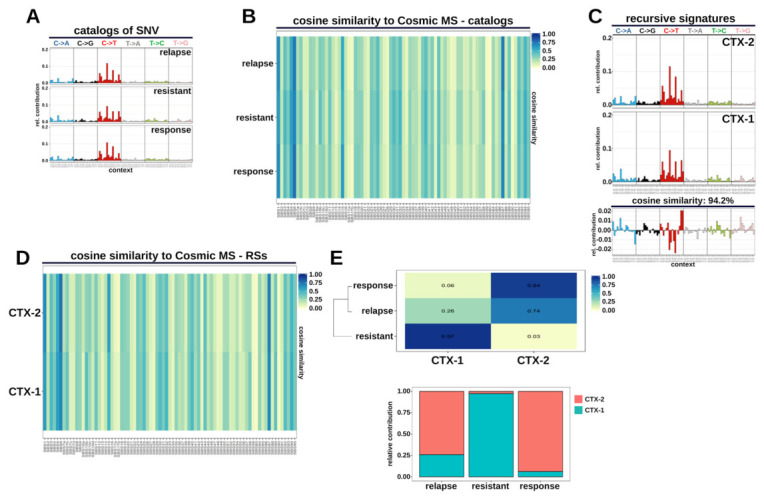
Prediction of response to induction CTX based on genomic data of AML-NCRI patients. (**A**) SNV catalogues of AML-NCRI patients grouped according to treatment response (relapse, resistant, and response groups), illustrating differences in mutational patterns across treatment response categories. (**B**) Cosine similarity of SNV catalogues to Cosmic SBSs MSs, indicating similarities and differences in mutational processes between groups. (**C**) CTX-1 and CTX-2, as extracted by NNMF and their cosine similarity to each other. (**D**) Cosine similarity of CTX-1 and CTX-2 to Cosmic SBSs MSs. (**E**) Contribution of CTX-1 and CTX-2 to reconstruction of mutational catalogues of AML-NCRI patient response groups, showing higher CTX-2 contribution in response and relapse groups, whereas the resistant group is predominantly characterised by CTX-1 contribution. The upper panel presents data as a heatmap, while the lower panel shows a corresponding bar chart representation.

Next, we subdivided patients according to their response to induction CTX into three groups: response, relapse, and resistant (Figure 4A). The cosine similarities of the Cosmic-SBS MSs to the new groups’ SNV catalogues were specific for each group, with a higher similarity of Cosmic-SBS MSs 10a and 15 to the response group’s catalogue, and a high similarity of the resistant group’s catalogue to Cosmic-SBS MSs 7b, 10b, 50, 51, and 58 (Figure 4B). NNMF extracted two RSs, CTX-1 and CTX-2, showing 94.2% similarity, which best differentiated the three patient groups (Figure 4C; Supplementary Figure S4). The cosine similarity of the two RSs to the Cosmic-SBS MSs is shown in Figure 4D. To investigate the clinical significance of CTX-1 and CTX-2, we analysed their contribution to the mutational data of patients’ response groups (Figure 4E). We could distinguish the resistant and response groups as they segregated into two distinct clusters. CTX-2 accounted for 74% and 94% of the mutational data in the response and relapse group catalogues, respectively, whereas CTX-1 contributed 97% of the mutational data in the resistant group catalogue (Figure 4E).

The clear distinction of the resistant group from the response/relapse groups indicates that the genome of these patients already encoded information which was predictive of response to induction CTX at the time of diagnosis.

### 3.5. The ML Algorithm RF Uses Encoded Genomic Information to Make Patient-Level Therapy Predictions

NNMF needs many somatic mutations (SNVs classified as SBSs) per patient to operate efficiently. In AML, obtaining a high number of mutations is challenging, even with WGS/WES, due to the relatively low mutational burden of the disease. As a result, NNMF typically requires genomic data from large retrospective datasets to accumulate sufficient mutations, which limits its applicability for prediction at the level of individual patients. We therefore investigated the potential of RF to analyse TS data from AML patients to make prospective predictions.

Initially, the patients were divided according to their response to induction CTX into two groups, responder and resistant, to make binary predictions. Relapse patients were added to the responder group because they had previously responded to induction CTX. The dataset was divided into a training set (70%) and a test set (30%). The training set was used to train the RF model, while the test set was used to evaluate its predictive performance. The input data for RF consisted of the matrix derived from the SNV catalogues of patients’ somatic mutations previously used in the MS analysis (See Methods).

An RF model fit comprising 1000 trees was then trained on the training set, and its predictive performance was evaluated on the test set using six parameters: Positive and Negative Prediction Value (PPV and NPV), sensitivity, specificity, accuracy, and balanced accuracy. The ROC-AUC incorporates both sensitivity and specificity, providing a valuable metric for evaluating the model’s ability to discriminate between classes. Higher AUC values indicate better model performance.

**Figure 5:**
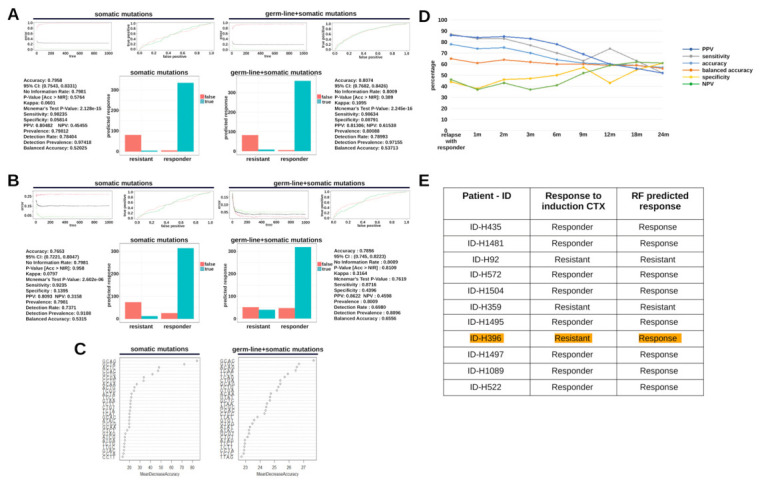
RF for prediction of therapy response using AML-NCRI TS data. (**A**) Unbalanced dataset: Somatic mutations versus germ line + somatic mutations. The uppermost figure on the left side of each mutation group shows the RF model fit performance over 1000 trees. The model fit indicates the error rates for different classes (red line for resistant class and green line for responder class) and out-of-bag samples (black) over mount of trees. The uppermost figure on the right side of each mutation group shows the ROC curve for the test set predictions. The lower figure in each mutation group represents a bar chart for the prediction performance of the test set. (**B**) Balancing data by SMOTE and the prediction of therapy response by RF. Somatic mutations versus germ line + somatic mutations RF model fit. The RF model fit plots on the left side of each mutation group indicate error for different classes (red line for resistant class and green line for responder class) and out-of-bag samples (black) over mount of trees. ROC curves for the test set predictions are shown on the right side of each mutation group. Bar charts show the prediction performance of the test set. (**C**) Variable importance: combined SBSs patterns that are decisive of class prediction. (**D**) Relapse and accuracy of RF. RF was applied repeatedly to the AML-NCRI dataset, with relapse patients classified as responders or resistant based on varying relapse time-point thresholds. Predictive performance was evaluated at each time point using six previously defined metrics. (**E**) Table showing 11 randomly selected patients used to assess the ability of RF to perform prospective predictions at the individual patient level. The highlighted patient was the only case that was incorrectly predicted by RF in this cohort.

The resulting model fit of the AML-NCRI somatic mutations was non-uniform, as shown in Figure 5A (the two lines, green and red, for responder and resistant, respectively, were non-parallel and widely separated from each other), indicating that the RF model fit could not identify the required SNV patterns specific to each response group in the training set. The model fit attained only 52% balanced accuracy in predicting induction CTX response in the test set patients (Figure 5A). In addition, the accuracy was highly skewed to the responder group.

### 3.6. Germline Mutations and Balanced Data Increased the Prediction Efficiency of RF

Germline mutations contribute to the pathogenesis of leukaemia as they relate to a person’s predisposition to develop AML [28], whereas somatic mutations reflect mutagen exposure [10]. To capture both, we constructed a “predisposition model” using unfiltered VCF files, yielding a dataset comprising germline (>99.99%) and somatic (<0.01%) mutations. SNV catalogues were used to generate a product matrix, which was split into a 70% training set and a 30% test set for RF model training and evaluation using six performance metrics. Incorporating germline mutations improved the prediction of the minority (resistant) group, increasing the NPV from 45% in the somatic-only RF model to 61% in the predisposition RF model (Figure 5A).

Prediction models such as RF often struggle with imbalanced datasets, as performance on the minority class is typically poor. This issue was relevant in our study, in which approximately 80% of patients achieved CR, and 20% were resistant. To address this imbalance, we applied the Synthetic Minority Over-sampling Technique (SMOTE) to the training sets for all subsequent RF analyses. This technique generates synthetic samples from existing minority-class instances without replicating data [29].

Balancing the AML-NCRI somatic mutation dataset slightly improved model performance, increasing specificity from 6% to 13% and balanced accuracy from 52% to 53% compared with the unbalanced model. However, the RF model fit, built on the balanced predisposition dataset, achieved considerable sensitivity (87%) and accuracy (79%), with increased specificity (44%), PPV (86%), NPV (46%), and balanced accuracy (65%). ROC curve analysis of the final RF model showed an AUC of 0.68 for test-set predictions. These results suggest the latest RF model detected the required prediction variables in the genomes of the balanced predisposition training set. It was able to train itself correctly, detecting 86% of responders and 46% of resistant patients in the test set (Figure 5B).

Comparison of the two RF models applied to the predisposition dataset, with and without training-set balancing, showed that the SMOTE-balanced model markedly improved prediction for the minority group, increasing specificity from 9% to 44% and balanced accuracy from 53% to 65% (Figure 5A, B).

A variable importance plot was generated to interpret the basis of the RF model’s fit and its predictions of patients’ outcomes (Figure 5C). These plots show the variables (specific SNV patterns) that contributed most to the prediction performance of the RF models trained on predisposition data and somatic mutation data. Higher values indicate the greater importance of a predictor in determining the patient outcome class [30]. In the predisposition model, variable contributions to model accuracy varied, with the GCAC mutation (C > A preceded by G and followed by C) showing the highest importance, followed by GTGC, ACAG, and TCAA (Figure 5C).

Finally, to determine how relapse patients should be classified and how their inclusion influences RF analysis, we performed nine separate RF analyses. In each RF prediction scenario, relapse patients were classified as responders or resistant according to different relapse time-point thresholds (1, 2, 3, 6, 9, 12, 18, and 24 months), as well as a model including all relapse cases with responders. Early relapse cases were assigned to the resistant group, whereas late relapse cases were assigned to the responder group. Following patient classifications, product matrices were generated from the patients’ SNV catalogues. Nine RF models were then applied to the differently classified versions of the dataset, and predictive performance was evaluated using six metrics. Among the different RF prediction models tested, classifying relapse patients within the responder group yielded the highest predictive performance (Figure 5D). These findings are consistent with our previous MS analysis, which similarly clustered relapse patients with the responder group (Figure 4E).

We next evaluated the potential of our RF model to prospectively predict individual patient responses to induction CTX. Eleven patients were randomly selected from the test set and analysed separately. As shown in Figure 5E, the RF model correctly predicted all 8 responders and 2 of 3 resistant patients, confirming its utility for patient-level response prediction.

## 4. Discussion

In this study, we demonstrated that TS data for 111 genes from 1552 AML patients provide sufficient genomic information for the bioinformatic prediction of therapy response and risk assessment. Comparisons of SNV patterns between the AML-NCRI and AML-SG TS datasets, as well as between TS and WES/WGS data, confirmed the reliability of the TS datasets and served as quality control for all subsequent analyses.

The first aim of this study was to explore the clinical significance of LDR using NNMF applied to genomic data of AML patients. In this approach, the number of patient groups in the input matrix had to exceed the number of RSs by at least one to enable clustering by NNMF. For example, the discrimination of four patient groups required no more than three RSs.

The similarity between the extracted RSs ranged from 86% to 94.2%, reflecting the fact that all analysed patients shared the same disease and underlying pathogenesis. Although NNMF produced highly similar RSs for a single disease, it still distinguished them as separate signatures. We analysed the datasets to extract RSs, intending to investigate whether the genomic data we used contained the information needed to predict the response to induction CTX and, ultimately, to perform risk assessment without incorporating clinical parameters. This objective differs from the identification of novel MSs, which are typically defined across many samples from diverse cancers to reveal defects in DDR mechanisms [10,11,31]. In that context, a similarity greater than 90% between two MSs would generally be considered too high to define a novel MS.

We introduced a novel isomorphism linking SNV catalogues from AML patients to signatures extracted via NNMF, which we term RSs for their iterative nature. Isomorphisms are the “Koebner phenomenon” or the relationship between DNA and the protein deciphered by the genetic code in the DNA [32,33]. As such, this mathematical framework enabled systematic mapping between patients’ genomic profiles and the extracted signatures, facilitating the stratification of patients with the same tumour. Using this approach, we examined clinically relevant endpoints, including response to induction CTX and risk stratification. Validation of our TS dataset using the independent TCGA WGS/WES datasets supported the robustness of our findings. Notably, RSs showed predictive value for both treatment response and risk stratification, indicating that this approach can reveal clinically relevant information embedded within tumour genomes. These findings highlight the potential of integrating mathematical modelling with genomic data to improve precision in AML management.

Our approach to identifying mutational patterns in the SNV catalogues and extracting RSs differs fundamentally from single-gene mutation analysis. In this framework, individual gene mutations represent only a subset of the broader SNV landscape, which also includes variants in regions not traditionally linked to disease pathogenesis. Importantly, any transformation of SNV data—such as grouping patients’ catalogues based on shared features—alters the input matrix for NNMF and, consequently, the derived RSs. This was reflected in our comparison of ELN 2017 and ELN 2022 risk classifications, where patient clustering shifted from intermediate/adverse to intermediate/favourable groups. These findings align with the reported improvements in OS and prognostic stratification for intermediate-risk patients under the updated ELN 2022 framework [27], thereby validating the robustness of our genomic analysis approach.

The cosine similarity between patient mutational profiles, RSs, and Cosmic-SBS MSs added a functional dimension to our analysis. Higher contributions of specific Cosmic-SBS MSs (e.g., smoking- or UV-associated) to an RS suggest that the corresponding DDR processes underlie that signature [31]. Consequently, the contribution of RSs to a patient group’s mutational catalogue can provide insight into the mutagenic processes shaping its mutational profile. In other words, the degree of cosine similarity between RSs and Cosmic-SBS MSs reflects the extent to which RSs capture the biological processes driving AML pathogenesis in patient groups with different clinical outcomes. Therefore, we can say that differences in DDR pathway involvement may reflect either subgroup-specific variation (e.g., response, resistance, and relapse) or fundamental mechanisms of leukaemogenesis. If DDR signalling defines AML subgroups, the contribution of Cosmic-SBS MSs to RSs or to groups’ SNV catalogues would vary across groups. Conversely, if these pathways drive leukaemogenesis, their contribution should be consistently high across all groups. These findings require experimental validation.

NNMF-generated RSs provided insight into AML disease history and progression. Still, they could not prospectively predict individual patient outcomes, as the signatures are mathematical constructs not directly comparable to single-patient data. The limitations of NNMF also include its dependence on a high number of somatic mutations to perform effectively, which is challenging in AML due to its low mutational burden. In addition, NNMF is restricted to somatic mutations and cannot incorporate germline variants, as it captures mutational processes that accumulate across the genome from the fertilised egg stage onwards without integrating inherited mutations [34]. Therefore, in this study, NNMF was used to confirm the presence of biologically relevant information and explore leukaemogenic mechanisms underlying treatment response and resistance, while alternative approaches were employed to leverage the encoded information for patient-level prediction.

To identify the most appropriate approach for extracting encoded information from patients’ genomes and utilising it to make prospective predictions at the single-patient level, several ML algorithms were investigated. Among the investigated tools, RF was the best algorithm to identify mutational patterns (specific SNV patterns) relevant to the clinical outcomes and use them to make prospective predictions of the outcome of novel patients. RF is a widely used ML algorithm to make predictions related to cancer, like progression and OS. In our study, we used it to predict the response to induction CTX in AML patients.

The problem we found with RF is that it is designed for balanced datasets. As clinical data and our dataset are often unbalanced, there is a need for a method to balance them, such as SMOTE. SMOTE is regarded as the standard approach for addressing imbalanced data in the field of ML, providing valuable information to improve the learning algorithm’s predictive performance for minority-class instances [22]. SMOTE greatly improved the predictive performance of the RF model, especially for the minority class, the resistant patients.

Integrating germline mutations improved our model’s predictive performance, suggesting their potential role in determining patient outcomes. This aligns with growing evidence highlighting the contribution of germline variation to the initiation, progression, and prognosis of haematological malignancies [28]. The clinical relevance of these mutations is increasingly recognised, as reflected by the inclusion of “myeloid neoplasms with germline predisposition” in the WHO classification [28]. Our final RF model achieved an AUC of 0.676, indicating predictive performance comparable to ELN 2022. It correctly predicted 46% of resistant patients, outperforming ELN 2022 risk stratification in identifying resistance within the adverse-risk group of the AML-NCRI cohort (see Supplementary Materials and Supplementary Table S1). These findings highlight the strong potential of our RF model to predict AML patients’ response to induction CTX.

Although risk assessment using RF predicted overall survival in AML patients better than the ELN 2022 risk stratification, with AUC values of approximately 0.72–0.75 [35], the model still has some limitations. These could be addressed through algorithm refinement, improvements in associated techniques such as SMOTE, and the expansion of the training dataset by including more patients and mutations. Additional challenges include the need to continuously adapt RF models to evolving, individualised therapies and to establish standardised, globally accepted bioinformatics workflows to generate homogeneous, high-quality datasets, particularly in rare diseases such as AML [36]. Our data also highlight a clear relationship between the number of mutations and prediction accuracy. While TS can accurately predict treatment response at the cohort level, reliable predictions for individual patients require WGS or WES to capture sufficient mutations.

## 5. Conclusions

In conclusion, our study demonstrates the potential of integrating genomic data with ML to improve risk assessment and predict response to induction CTX in AML. NNMF revealed that mutational catalogues contain sufficient information for risk stratification and prediction of therapy response. RF enabled accurate individual-level predictions and identified specific sequential mutational patterns influencing patients’ outcomes. These findings support the feasibility of integrating genomic data into clinical decision-making for personalised AML therapy.

## Data Availability

The AML NCRI dataset’s raw sequencing data is deposited in the European Genome-Phenome Archive (EGA; accession EGAS00001000570) and is available at: https://ega-archive.org/studies/EGAS00001000570 (accessed on 4 June 2026). The AML-SG dataset was derived from the data repository [15] and the European Genome-Phenome Archive (EGA; accession EGAS00001000275) and is available at: https://ega-archive.org/studies/EGAS00001000275 (accessed on 4 June 2026). The TCGA AML data were obtained from The Cancer Genome Atlas (TCGA), which is available at https://www.cancer.gov/ccg/research/genome-sequencing/tcga (accessed on 4 June 2025).

## Supplementary Materials

The following supporting information can be downloaded at: https://www.mdpi.com/article/10.3390/cancers18121925/s1, Supplementary Information I and II: Supplementary Results and Supplementary Materials and Methods; Figure S1: Bioinformatic pipeline for variant calling and annotation of the AML-NCRI predisposition dataset; Figure S2: Risk stratification analysis of the TCGA and AML-NCRI datasets and determination of the optimal number of mutational signatures; Figure S3: Prognostic performance of ELN2017 and ELN2022 risk stratification systems in the AML-NCRI cohort; Figure S4: NNMF survey rank analysis following exclusion of early deaths (ED) from the AML-NCRI cohort; Table S1: The estimated prediction efficiency of the ELN2022 risk stratification system for the response to induction CTX in the AML-NCRI cohort.
