# Supplementary material for: Mutational Signatures and Machine Learning for Risk Stratification of Acute Myeloid Leukaemia Based on Targeted Sequencing Data
Source: Cancers (Basel). 2026 Jun 12;18(12):1925. doi: 10.3390/cancers18121925 (PMC13297301; doi:10.3390/cancers18121925)
Supplement: Supplementary file 1 [file cancers-18-01925-s001.zip › Suppl-Information-II- figures and tables.pdf]

Mutational Signatures and Machine Learning for Risk Stratification of Acute Myeloid Leukaemia Based on Targeted Sequencing Data

Heba Elhaddad[1,2,3,*], Claudia Chiriches[1,2], Shuvro Prokash Nandi[4,5], Patrick van Eijk[4], Amanda Gilkes[1,2], Katie Watts[4], Amy Houseman[4], Charlotte S. Wilhelm-Benartzi[6], Oliver Gerhard Ottmann[1,2], Simon H. Reed[4,*], and Martin Ruthardt[1,2]

[1] Division of Cancer and Genetics, Section of Haematology, School of Medicine, Cardiff University, Cardiff CF14 4XN, UK.

[2] Experimental Cancer Medical Centre (ECMC), School of Medicine, Cardiff University, Cardiff CF14 4XN, UK.

[3] Clinical Pathology Department, Faculty of Medicine, Mansoura University, Mansoura 35516, Egypt.

[4] Division of Cancer and Genetics, School of Medicine, Cardiff University, Cardiff CF14 4XN, UK.

[5] Department of Cellular and Molecular Medicine, University of California San Diego, La Jolla, CA 92093, USA

[6] Centre for Trials Research, School of Medicine, Cardiff University, Cardiff CF14 4XN, UK.

* Correspondence: elhaddadha@cardiff.ac.uk (H.E.); reedsh1@cardiff.ac.uk (S.H.R.); Tel.: +44-(0)29-2074-4194

Running title: Bioinformatics in acute myeloid leukaemia

Supplementary Figures

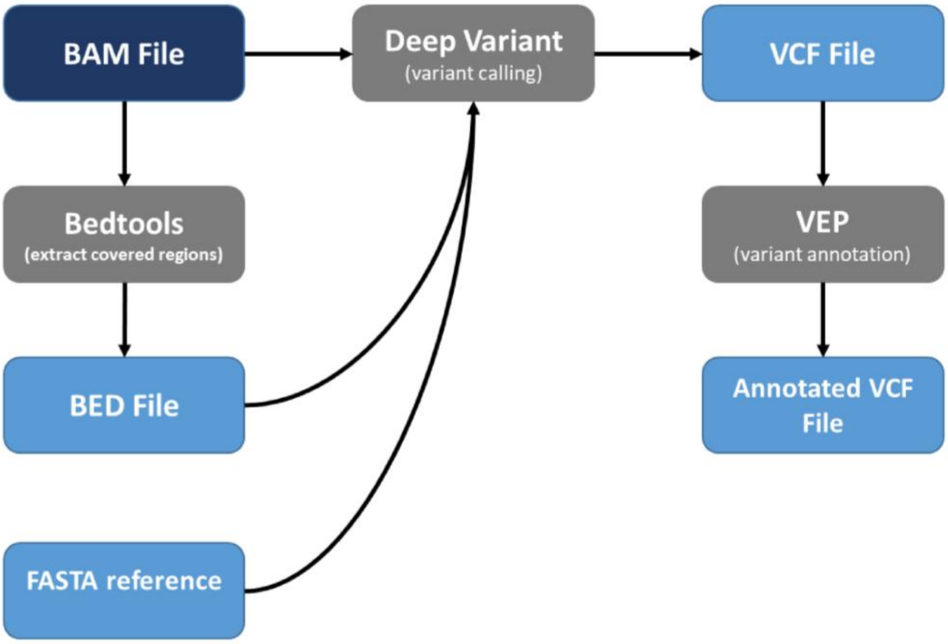

Supplementary Figure S1. Bioinformatic pipeline for variant calling and annotation of the AML-NCRI predisposition dataset. Sequencing BAM files were processed using BEDtools to identify covered regions and generate BED files. Variant calling was performed using DeepVariant with the corresponding reference genome, generating VCF files that were subsequently annotated using VEP to produce annotated variant datasets. Abbreviations: BAM, Binary Alignment Map (raw sequencing files); BEDtools, software suite for processing BED files; BED, Browser Extensible Data file containing genomic coordinates and annotations; DeepVariant, deep neural network-based variant calling pipeline; VCF, Variant Call Format file storing sequence variations; VEP, Variant Effect Predictor annotation software.

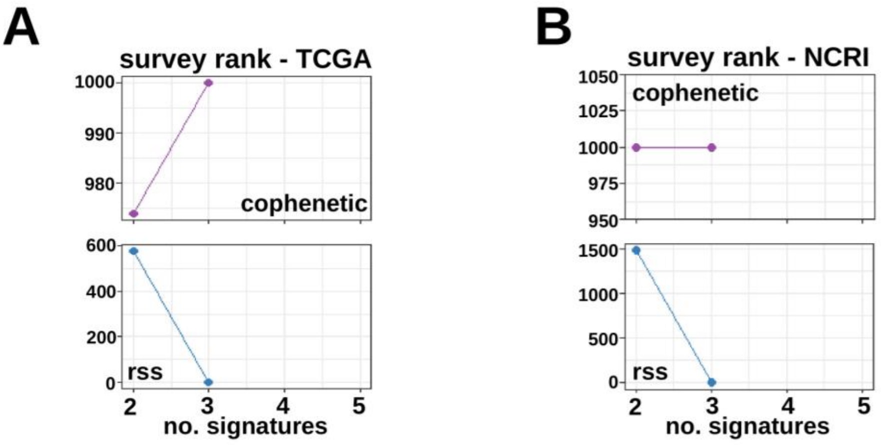

Supplementary Figure S2. Rank survey analysis for the risk stratification of the TCGA and AML-NCRI datasets. Determination of the optimal number of mutational signatures. **(A)** NNMF survey rank analysis for the extraction of RSs from the TCGA dataset. **(B)** NNMF survey rank analysis for the extraction of RSs from the AML-NCRI dataset. The cophenetic coefficient and residual sum of squares (rss) were used to determine the optimal number of signatures that could be extracted from each cohort to best describe the risk groups. The cophenetic correlation coefficient measures how faithfully clustering approaches preserve pairwise distances and, therefore, dendrogram structures. A high cophenetic coefficient indicates stable model reproducibility. The rss represents the reconstruction error, where a lower reconstruction error indicates that the selected number of signatures provides a more accurate representation of the original cohort genome catalogues. The optimal number of signatures was determined as the rank with the highest cophenetic coefficient that, when increased by one additional signature, did not result in a substantial reduction in the rss.

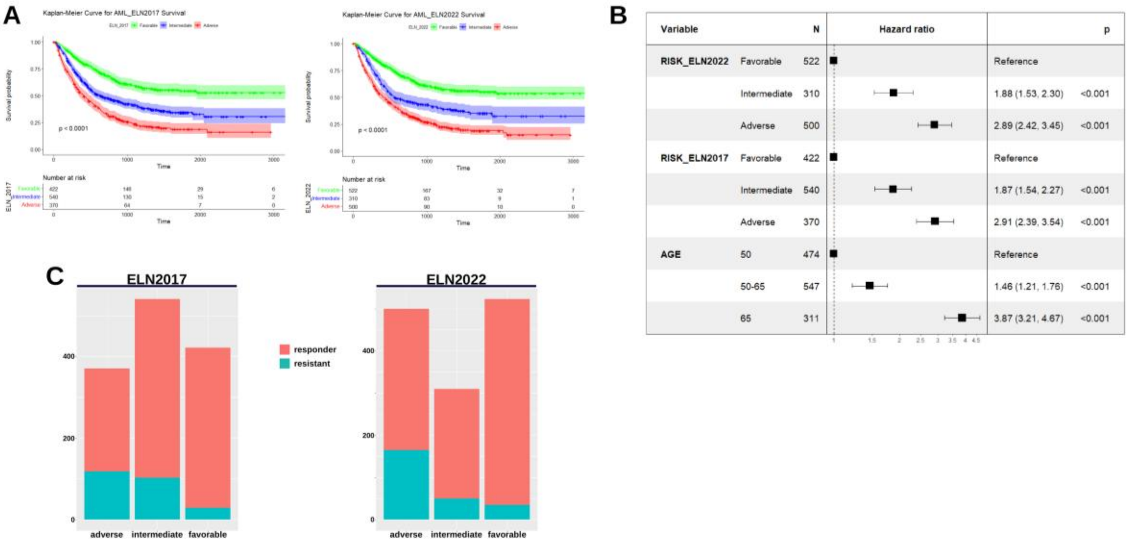

Supplementary Figure S3. Prognostic performance of ELN2017 and ELN2022 risk stratification systems in the AML-NCRI cohort. (A) Kaplan–Meier (KM) curves comparing overall survival (OS) among AML-NCRI patients classified into three risk groups according to ELN2017 and ELN2022, respectively. **(B)** Univariate Cox regression analysis showing hazard ratios and 95% confidence intervals for ELN2017, ELN2022, and age groups. **(C)** The ability of ELN risk stratification to predict response to induction chemotherapy (CTX) in the AML-NCRI cohort. The figure shows complete remission (CR; responders) and resistance rates across the three ELN risk groups according to ELN2017 and ELN2022 classifications.

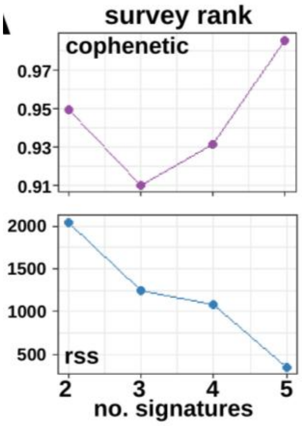

Supplementary Figure S4. NNMF survey rank analysis following exclusion of early deaths (ED) from the AML-NCRI cohort. NNMF survey rank analysis to determine the optimal number of signatures to be extracted from the AML-NCRI dataset following the exclusion of patients classified as ED.

Supplementary Tables

	**Positive predicted (favourable, intermediate)**	**Negative predicted (adverse)**	
Actual positive (responder)	488+260=748 (TP)	335 (FN)	**Sensitivity = 69%**
Actual negative (resistant)	34+ 50 = 84 (FP)	165 (TN)	**Specificity = 66%**
	**PPV = 89.9% (Precision)**	**NPV = 33%**	**Accuracy = 68.5%**

Supplementary Table S1. The estimated prediction efficiency of the ELN2022 risk stratification system for the response to induction CTX in the AML-NCRI cohort.
