# Supplementary material for: Mutational Signatures and Machine Learning for Risk Stratification of Acute Myeloid Leukaemia Based on Targeted Sequencing Data
Source: Cancers (Basel). 2026 Jun 12;18(12):1925. doi: 10.3390/cancers18121925 (PMC13297301; doi:10.3390/cancers18121925)
Supplement: Supplementary file 1 [file cancers-18-01925-s001.zip › Suppl-Information-I.pdf]

Mutational Signatures and Machine Learning for Risk Stratification of Acute Myeloid Leukaemia Based on Targeted Sequencing Data

Heba Elhaddad[1,2,3,*], Claudia Chiriches[1,2], Shuvro Prokash Nandi[4,5], Patrick van Eijk[4], Amanda Gilkes[1,2], Katie Watts[4], Amy Houseman[4], Charlotte S. Wilhelm-Benartzi[6], Oliver Gerhard Ottmann[1,2], Simon H. Reed[4,*], and Martin Ruthardt[1,2]

[1] Division of Cancer and Genetics, Section of Haematology, School of Medicine, Cardiff University, Cardiff CF14 4XN, UK.

[2] Experimental Cancer Medical Centre (ECMC), School of Medicine, Cardiff University, Cardiff CF14 4XN, UK.

[3] Clinical Pathology Department, Faculty of Medicine, Mansoura University, Mansoura 35516, Egypt.

[4] Division of Cancer and Genetics, School of Medicine, Cardiff University, Cardiff CF14 4XN, UK.

[5] Department of Cellular and Molecular Medicine, University of California San Diego, La Jolla, CA 92093, USA

[6] Centre for Trials Research, School of Medicine, Cardiff University, Cardiff CF14 4XN, UK.

* Correspondence: elhaddadha@cardiff.ac.uk (H.E.); reedsh1@cardiff.ac.uk (S.H.R.); Tel.: +44-(0)29-2074-4194

Running title: Bioinformatics in acute myeloid leukaemia

Supplementary Results

Evaluation of ELN2017 and ELN2022 on the AML-NCRI patients. According to Lachowiez et al. (2023), 42% of patients classified as intermediate risk by ELN2017 were reclassified into the favourable or adverse risk groups in ELN2022. We analysed overall survival (OS) to evaluate the impact of the ELN2022 updates in the AML-NCRI cohort. Kaplan–Meier curves showed an improved OS curve of the intermediate-risk group in ELN2022 compared with ELN2017 (Supplementary Figure S2A). Univariate Cox regression analysis of the three risk groups did not demonstrate a significant change from ELN2017 to ELN2022 (Supplementary Figure S2B). Next, we examined the distribution of patients across the risk groups in both classifications. Supplementary Figure S2C illustrates the redistribution of intermediate-risk patients into the favourable and adverse-risk groups.

The efficiency of ELN2022 in predicting response to induction chemotherapy

To estimate the additional value of our ML approach beyond current AML risk stratification systems, we compared its predictive performance with that of ELN2022. Therefore, we assessed the theoretical ability of ELN2022 to predict response to induction chemotherapy (CTX) in AML-NCRI patients.

To ensure direct comparability with the ML models, patients were classified into two response categories: responders and resistant patients. Patients who relapsed after induction CTX were included in the responder group, as they initially achieved a response to treatment.

To estimate the predictive performance of ELN2022, we assumed that the risk stratification system would correctly classify:

- Adverse-risk patients, who are expected to have a poor prognosis, as resistant to induction CTX (negative prediction class).
- Favourable- and intermediate-risk patients, who are expected to have a better

prognosis, as responders to induction CTX (positive prediction class).

The actual numbers of patients in each ELN2022 risk group were then compared with their induction CTX response status. The numbers of responder patients classified as adverse-, intermediate-, and favourable-risk were 335, 260, and 488, respectively. The corresponding numbers of resistant patients were 165, 50, and 34.

These data were subsequently arranged in a contingency table to calculate the same prediction performance metrics used for the ML models, employing standard accuracy assessment formulas. The resulting performance measures were then compared with those obtained using the RF model.

The RF model previously achieved an accuracy, sensitivity, specificity, positive predictive value (PPV; precision), and negative predictive value (NPV) of 78.56%, 87.16%, 44.00%, 86.00%, and 46.00%, respectively, for predicting AML patient response to induction CTX. Comparison with the results presented in Supplementary Table S1 showed that the RF model achieved higher accuracy, sensitivity, and NPV than ELN2022, whereas ELN2022 demonstrated higher specificity and PPV for predicting response to induction CTX.

From BAM to VCF files: The workflow for generating the predisposition model.

The filtering of NGS data for the analysis of malignant diseases is primarily designed to identify somatic mutations. Consequently, variants associated with an individual's genetic predisposition to disease may be excluded during the filtering process. To investigate the contribution of predisposition variants to RF-based prediction of therapy response, we modified the filtering strategy and generated VCF files containing a broader set of variants. Supplementary Figure S4 illustrates the workflow used to develop the predisposition model in AML-NCRI patients.

Supplementary Materials and Methods

Survey rank. No consensus method for automatically evaluating the optimal factorisation rank has been found. Therefore, matrix decomposition was performed iteratively across a range of factorisation ranks, and multiple quality metrics were calculated for each rank. The best rank was chosen according to the highest cophenetic correlation coefficient with non-sharp declining rss (residual sum of squares). The rss explained whether a variance helps compare the different models' performance and ability to reproduce the original target matrix accurately. In addition, the number of extracted signatures was constrained to be lower than the number of predicted classes.

Kaplan Meier curves (KM). KM analysis was performed on the AML-NCRI dataset to compare OS among the three risk groups for each ELN classification using the log-rank test P-value. Analysis was done in R using "Survminer," "Survival," and "gtsummary" packages. Both ELN2017 and ELN2022 classifications showed statistically significant differences in OS among the three risk groups.
