# Supplementary figures and images for: Mutational Signatures and Machine Learning for Risk Stratification of Acute Myeloid Leukaemia Based on Targeted Sequencing Data
Source: Cancers (Basel). 2026 Jun 12;18(12):1925. doi: 10.3390/cancers18121925 (PMC13297301; doi:10.3390/cancers18121925)

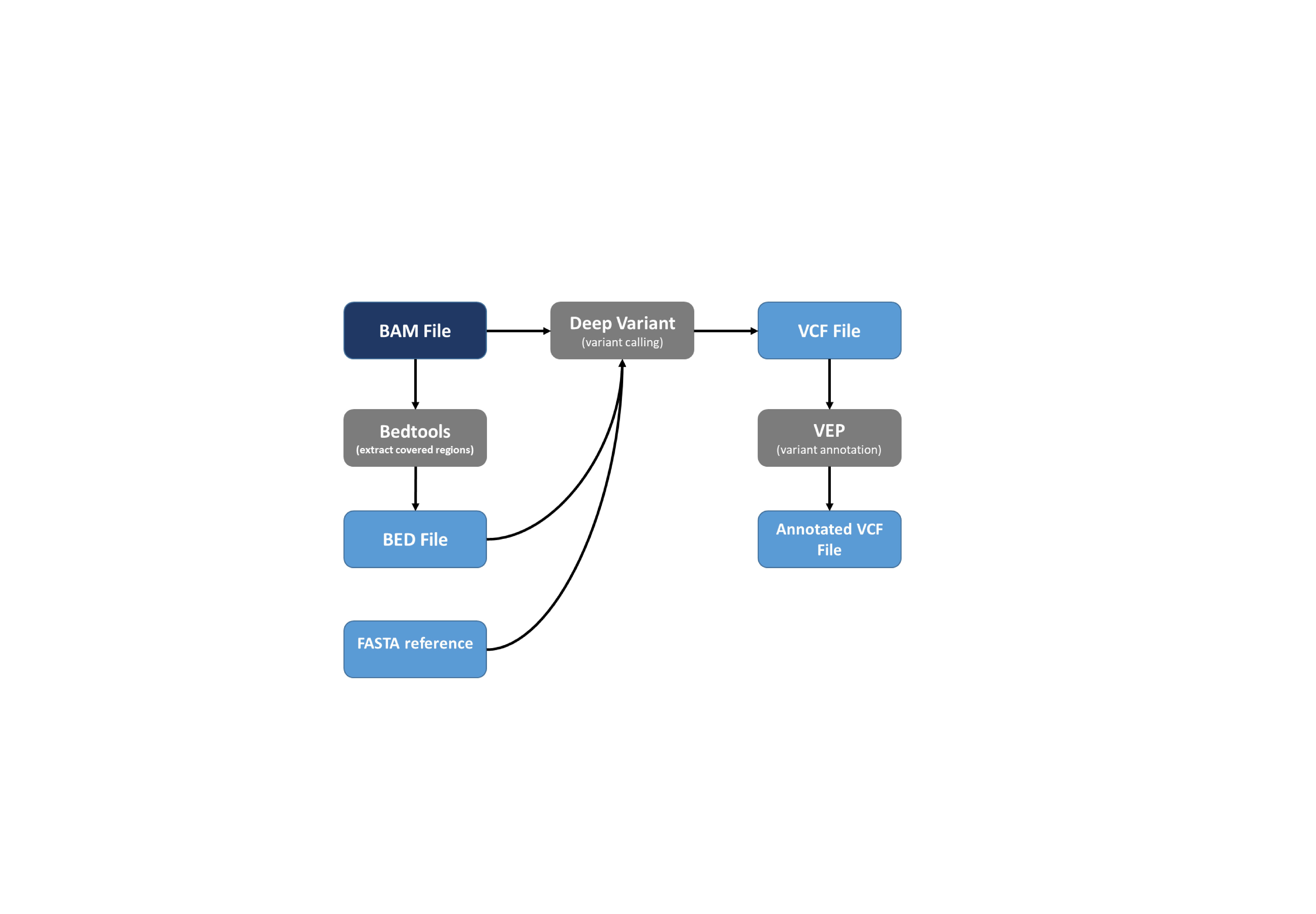

Supplement: Supplementary file 1 [file cancers-18-01925-s001.zip › supplementary figures 600dpi/Suppl-figure 1.jpg]

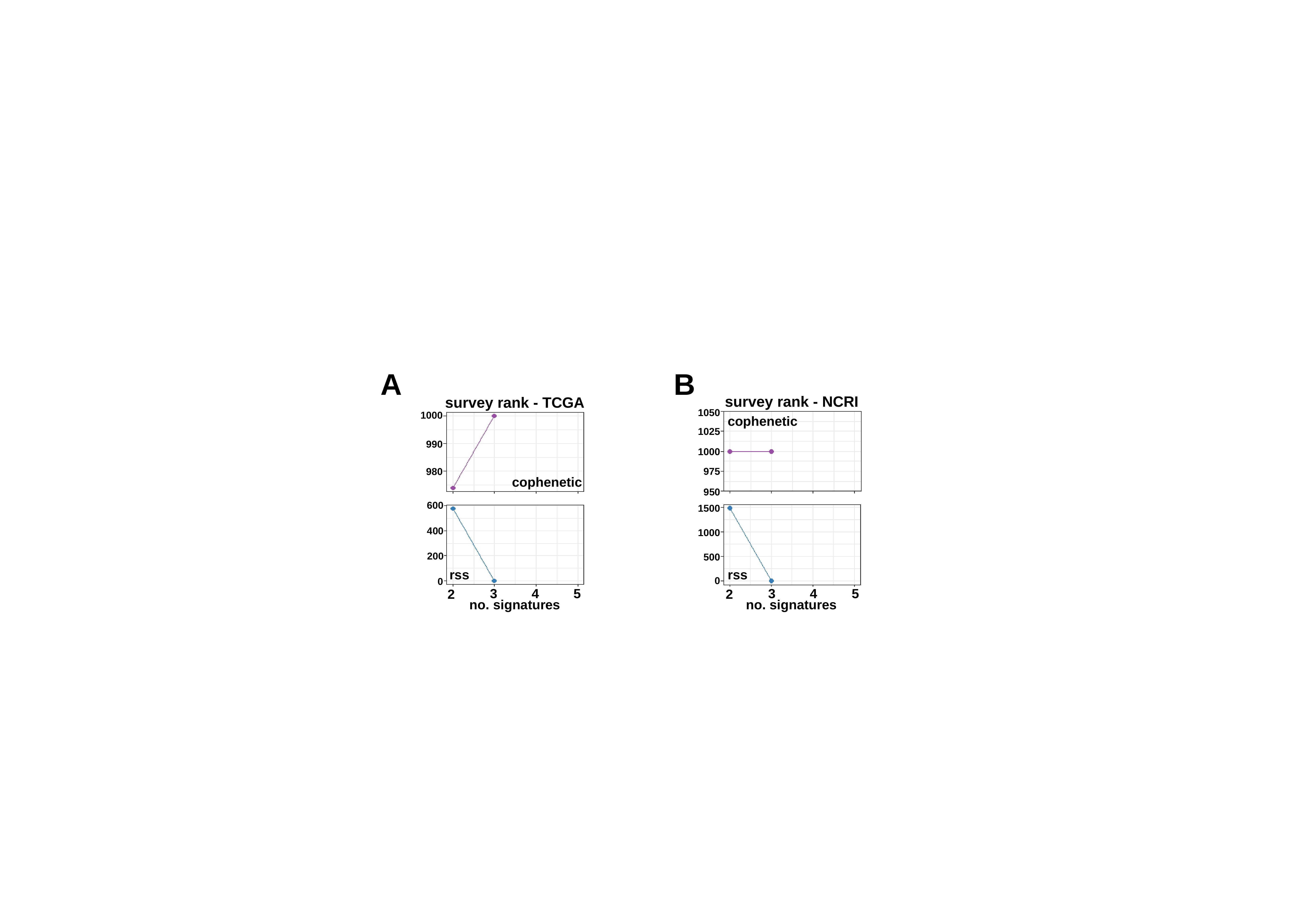

Supplement: Supplementary file 1 [file cancers-18-01925-s001.zip › supplementary figures 600dpi/Suppl-figure 2.jpg]

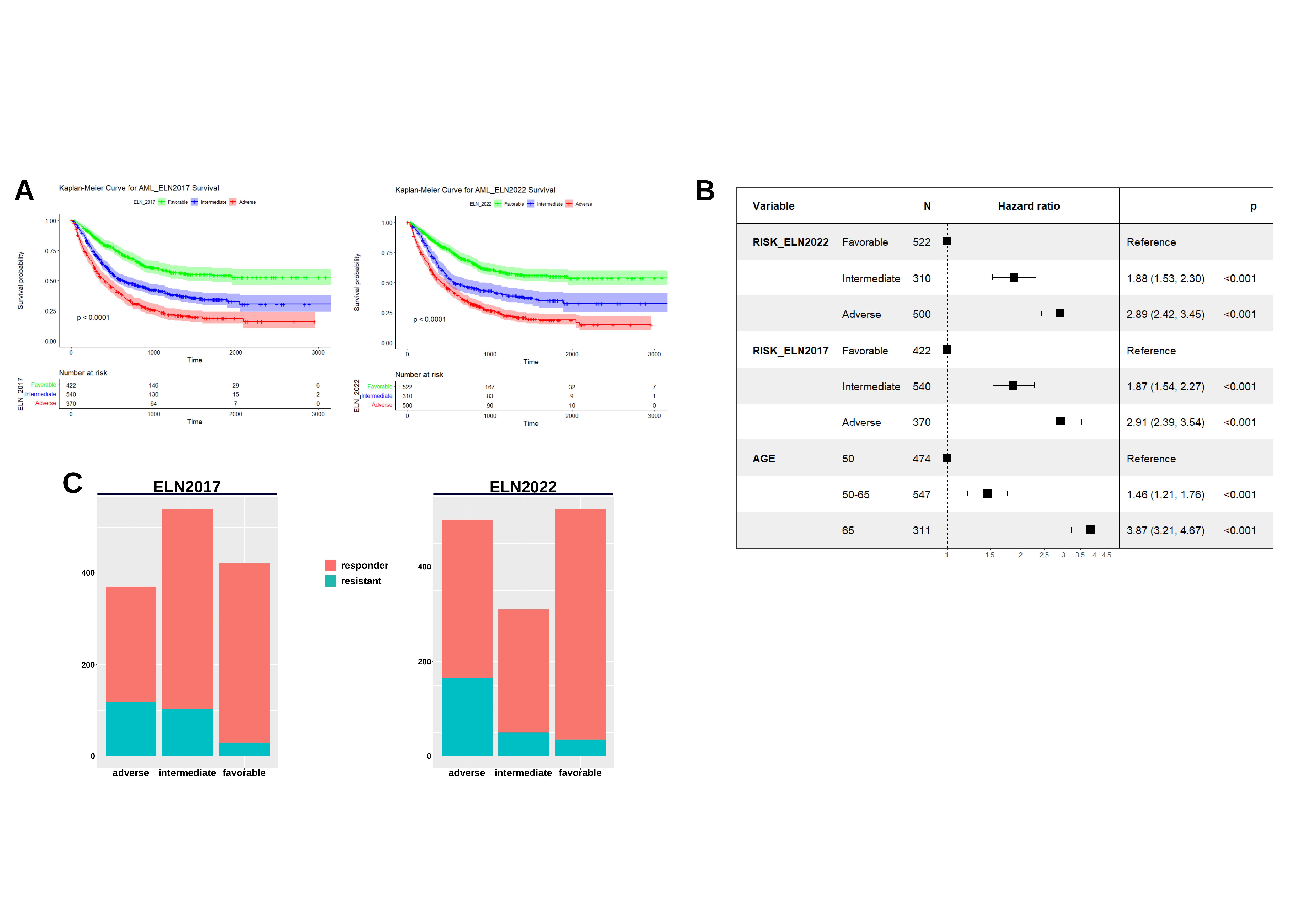

Supplement: Supplementary file 1 [file cancers-18-01925-s001.zip › supplementary figures 600dpi/Suppl-figure 3.jpg]

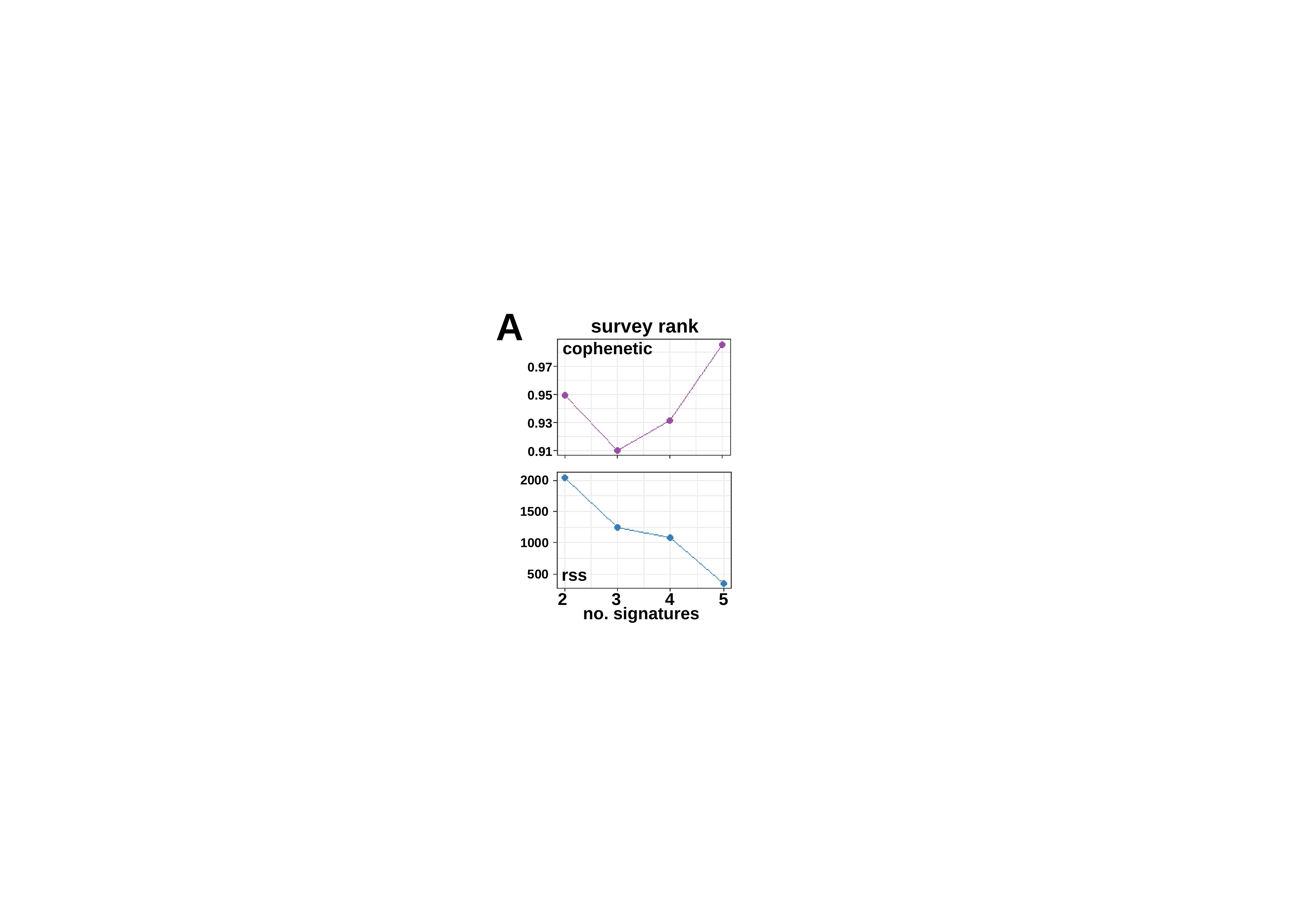

Supplement: Supplementary file 1 [file cancers-18-01925-s001.zip › supplementary figures 600dpi/Suppl-figure 4.jpg]
